# From Diversion to Permanence: Trends in Ostomy Creation in Rectal Cancer Surgery

**DOI:** 10.3390/jcm14061913

**Published:** 2025-03-12

**Authors:** Alice Jo, Matthew Z. Wilson

**Affiliations:** 1 Department of Surgery, Dartmouth-Hitchcock Medical Center, Lebanon, NH 03766, USA; matthew.z.wilson@hitchcock.org; 2 Geisel School of Medicine at Dartmouth, Hanover, NH 03755, USA

**Keywords:** rectal cancer surgery, temporary ostomy, permanent ostomy, diverting ostomy, sphincter preservation, ostomy complications

## Abstract

Rectal cancer surgery has undergone transformative advancements over the past few decades, evolving from radical, high-morbidity procedures to more refined techniques focused on both oncological outcomes and the preservation of anorectal function. This review provides a brief overview of the history of rectal cancer surgery, highlighting key innovations in imaging, neoadjuvant therapy, and minimally invasive techniques that have significantly reduced the need for permanent and temporary ostomies. Additionally, the current indications for both permanent and temporary ostomies are reviewed, including a discussion of associated complications, such as non-reversal, parastomal hernias, stomal prolapse, stenosis, and skin-related issues, along with strategies and techniques to mitigate these complications. This review underscores the importance of ongoing innovation and individualized surgical planning to enhance patient outcomes in rectal cancer care by understanding the historical context, contemporary practices, and associated challenges.

## 1. Introduction

Rectal cancer surgery has seen remarkable advances over the decades, yet the decision to utilize an ostomy remains a critical aspect of personalized rectal cancer care. Ostomies, whether temporary or permanent, play a pivotal role in ensuring optimal oncologic outcomes, reducing fatal postoperative complications, and maintaining patient quality of life. The decision to perform ostomies is nuanced, balancing the need for disease control with the goal of functional preservation.

Historically, permanent end colostomies were considered the standard for managing rectal tumors. This approach prioritized oncologic safety by ensuring complete tumor resection. However, advancements in our understanding of the spread of rectal cancer, imaging technologies, neoadjuvant therapies, and minimally invasive surgical techniques have significantly shifted the treatment paradigms. Accurate preoperative anatomic assessment from rectal magnetic resonance imaging (MRI), tumor downstaging through chemoradiation, and the precision offered by robotic-assisted surgeries have paved the way for sphincter-preserving procedures, reducing the reliance on permanent or temporary ostomies.

Despite these innovations, temporary or permanent ostomies remain crucial in many cases of rectal cancer, influenced by patient-specific factors and overall treatment goals. The current review explores up-to-date indications for temporary and permanent ostomies in rectal cancer care. Additionally, the review examines complications related to ostomy creation and reversal as well as emerging technical strategies to reduce ostomy-related complications. Ultimately, this review aims to provide a comprehensive understanding of the role of ostomies in personalized rectal cancer care, highlighting the delicate balance between surgical innovation and patient-centered care.

## 2. A Brief History of Rectal Cancer Surgery

### 2.1. Evolution from Abdominoperineal Resection to Sphincter-Preserving Techniques

Traditionally, treatment of rectal cancer required resecting the rectal tumor and perineum which resulted in a permanent end colostomy. The emergence of anesthesiology in the early 19th century revolutionized surgery by enabling muscle relaxation during laparotomy, making extensive operations feasible [1,2]. In 1874, Swiss surgeon Theodor Kocher introduced trans-sacral resection with coccygectomy to improve exposure of the rectum for resection, a technique further refined by Paul Kraske [1,2,3]. The term “radical abdominoperineal resection (APR)” was first introduced by English surgeon Wiliam Ernest Miles in 1908 in The Lancet [1,2]. Miles described a procedure that involved the extensive excision of the rectum, anal canal, levator ani, and draining lymph nodes [1,2]. Initial studies using this technique reported a 42% mortality rate and a 90% recurrence rate [1,2].

The mid-20th century saw significant development in sphincter-sparing techniques for upper- and mid-rectum rectal cancer. Around 1940, Babcock and Bacon separately introduced pull-through techniques, where the rectum was resected and the mobilized colon was pulled outside the anus to create an anastomosis [4,5,6], and Babcock added a diverting colostomy to protect the distal anastomosis [4]. The basis for the modern transabdominal low anterior resection (LAR) is credited to Claude Dixon of the Mayo Clinic, who described a rectal resection in the abdominal cavity and two-layer anastomosis for upper rectal cancer in 1948 [1,6].

Rectal cancer surgery reached a significant milestone in the 1980s when Heald described total mesorectal excision (TME), which involved en bloc removal of the mesorectum, vascular and lymphatic structures, fatty tissue, and mesorectal fascia as a “tumor package” through sharp dissection [2,6,7]. Heald’s insight was that incomplete and inconsistent resection of the “danger zone” within the mesorectum contributed to the wide variation in reported local recurrence rates [8]. Sharp dissection of the avascular alveolar plane between the presacral and mesorectal fascia, known as the “holy plane”, became the gold standard in rectal cancer surgery [8], reducing the local recurrence rates after rectal cancer surgery to 3.6% [6,7].

### 2.2. A Brief History of Ostomy Surgery and Care

Unlike the broader evolution of rectal cancer surgery, the concepts and techniques of ostomies have remained largely consistent over time. Early stomas were spontaneous fistulas that formed after bowel perforation, with surgeons observing improved patient survival [9]. One of the earliest documented end colostomies was performed in the late 18th century on a 3-day-old infant with an imperforate anus, laying the groundwork for end colostomy in cases of obstructive cancer and for the development of APR in rectal cancer surgery [10]. In the latter half of the 20th century, surgeons found that proximal diverting colostomy could protect distal anastomosis in LAR and prevent pelvic sepsis [2,9,11]. In 1888, a support rod was first introduced to prevent retraction of the loop stoma, which allowed for better diversion of the fecal stream [9,10].

One of the first accounts of surgical ileostomy involved bringing the cut edge of the ileum out of the skin and letting the distal end of the stoma slough off for “self-maturation” [10]. In the 1950s, Bryan Brooke pioneered everting the bowel mucosa to create an end ileostomy, reducing skin breakdown complications and retraction associated with spontaneous maturation [12]. A modern loop ileostomy, as we know it today, was first described by Turnbull and Weakley in the late 1960s [13], which gained popularity for its technical simplicity in creation and reversal.

Recognizing the challenges patients faced in managing ostomies, Turnbull recruited one of his patients with an ileostomy, Norma N. Gill, to provide education and support for individuals undergoing ostomy surgeries in 1958 [10,14]. They also partnered with manufacturers to develop improved pouching systems, established routine preoperative stoma site marking, created support systems, and initiated formal enterostomal therapy training programs [14]. The enterostomal therapists developed a national association within a few years, which evolved into the Wound, Ostomy, and Continence Nurses (WOCN) Society [14].

## 3. Modern Innovations Minimizing Ostomy Requirements in Rectal Cancer Care

### 3.1. Reduction in Recommended Distal Resection Margin

Improvements in our understanding of tumor biology have enabled the reduction in the recommended distal margin for rectal cancer from 5 cm to 1 cm [15,16]. This shift has facilitated more sphincter-preserving surgeries, reducing the need for permanent end colostomies. Recent studies have explored the outcomes of distal resection margins (DRM) smaller than 1 cm [17,18,19]. A retrospective analysis of 415 rectal cancer pathological specimens found no significant differences in local recurrence or survival rates between distal resection margins of 0.1–0.5 cm and 0.5–1 cm [17]. Some examples of ultra-low rectal cancer anastomosis include intersphincteric resection with handsewn coloanal anastomosis [20] and Turnbull–Cutait pull-through anastomosis [21]. However, their utility is constrained by suboptimal functional outcomes rather than limitations in oncologic outcomes.

### 3.2. Improvements in Magnetic Resonance Imaging Techniques

Rectal MRI has become indispensable in rectal care management, as modern rectal cancer care heavily relies on its use for surgical planning. Beyond providing tumor-node-metastasis (TNM) staging, rectal MRI can provide a precise evaluation of circumferential resection margins (CRM), detection of extramural vascular invasion, and identification of tumor deposits [22]. Several technical improvements in rectal MRI in the last two decades include thin-section MRI [23], high-resolution surface coil and gradient coil systems [24], and the use of rectal contrast agents or bowel paralytics in some institutions [25].

In 2006, the MERCURY (Magnetic Resonance Imaging and Rectal Cancer Equivalence Study) trial, a multicenter multidisciplinary collaboration, demonstrated that high-resolution MRI had a 92% specificity in predicting clear CRM, highlighting its reliability in assessing CRM involvement preoperatively [26]. The trial established that MRI could stratify patients into “high-risk” and “low-risk” categories, allowing tailored treatment approaches in considering neoadjuvant therapy before surgery [26,27]. Similarly, a prospective cohort study demonstrated that a tumor-free margin of at least 1 mm could be predicted with MRI [28]. The MERCURY II trial in 2014 showed that high-resolution MRI could accurately assess the relationship between low rectal tumors and the anal sphincter: the odds ratio of pathological CRM rates was 5.5 (95% CI, 2.3–13.3) for “unsafe” MRI-predicted surgical resection planes compared to the “safe” ones [29].

The advances in rectal MRI technology solidified its role as an essential tool for accurate staging, CRM assessment, identification of high-risk features, and therapy response evaluation, enabling more precise surgeries, higher rates of sphincter preservation, and better oncologic outcomes for personalized rectal cancer care.

### 3.3. Neoadjuvant Chemotherapy and Radiation Therapy

Neoadjuvant therapy has made it possible for significant downstaging of low rectal cancers, allowing patients to undergo sphincter-sparing surgery when they would have otherwise not been candidates. Neoadjuvant therapy for patients with distal cT2-T4 or node-positive rectal cancer has increased the rate of sphincter preservation [20,30,31,32]. One trial found that patients who received preoperative chemoradiation (CRT) were twice as likely to undergo a sphincter-sparing operation (39 vs. 19%) compared to those who received postoperative CRT [33].

In the past two decades, total neoadjuvant therapy (TNT) for locally advanced rectal cancer has opened the doors to “watch-and-wait” strategies for patients with a complete clinical response (cCR), conferring an opportunity for organ preservation by avoiding surgery altogether in many cases [34,35,36]. In the OPRA (Organ Preservation in Rectal Adenocarcinoma) trial, nearly half of the patients treated with TNT avoided TME surgery entirely, with a higher rate of cCR in the consolidation TNT group than the induction TNT group (51% vs. 63%) [36]. Tumor regrowth occurred in 25% of “watch-and-wait” patients, with the majority occurring within the first two years, but those who required delayed surgery after regrowth were comparable to those who had immediate surgery [36].

The role of neoadjuvant therapy in reducing the rate of temporary diverting ostomies is less clear. A meta-analysis revealed that for patients who do not achieve a cCR following a TNT regimen, TNT did not significantly reduce the rate of ileostomy compared to the standard neoadjuvant regimen [37]. However, TNT may be associated with earlier ileostomy reversal: a retrospective cohort study of 24 patients showed that the median times to reversal were 3.6 months for the TNT group versus 6.9 months for the traditional sequence group (47% reduction, *p* = 0.001) [38]. The unchanged ileostomy rate with TNT likely stems from radiation-induced changes in the rectum, leading surgeons to favor the use of diverting ostomies to safeguard distal anastomoses. Consequently, radiation-free neoadjuvant regimens are being explored. In a phase III trial comparing neoadjuvant chemotherapy with CAPOX to TNT in patients with locally advanced rectal cancer and uninvolved mesorectal fascia, the ileostomy rates were significantly lower in the CAPOX group (52.0% vs. 64.6%, *p* = 0.008) [39].

Neoadjuvant chemoradiation facilitates tumor downstaging, enhances margin clearance, reduces the risk of local recurrence, and enables a “watch-and-wait” strategy for select patients. Together, these benefits improve sphincter preservation and decrease the need for permanent colostomies. Its role in reducing the rate of temporary diverting ostomies has not been established.

### 3.4. Minimally Invasive Techniques

Minimally invasive techniques, particularly robotic-assisted surgery, have improved rectal cancer surgery by enhancing visualization of the surgical field, enabling more precise dissection with greater instrument flexibility, and facilitating lower anastomoses [40]. Compared to laparoscopic LAR, robotic-assisted LAR has shown lower rates of Clavien-Dindo grade III–V severe complications (OR = 0.69, 95% CI 0.53–0.90) [41]. Additionally, robotic-assisted LAR has been associated with a lower permanent stoma rate than laparoscopic LAR and transanal TME (21.3% robotic, 40.1% laparoscopic, 25.6% transanal, *p* < 0.001) [42]. The cause of lower permanent stoma rate in robotic-assisted LAR seems to be multifactorial, rather than due to less anastomotic leak. Although the use of robotic staplers has been associated with a significantly higher rate of negative air leak tests compared to manual or powered circular staplers in LAR [43], this finding has not translated into clinical outcomes, as several meta-analyses have shown no significant difference in anastomotic leak rates between robotic-assisted and laparoscopic LAR or intersphincteric resection (ISR) [41,44,45].

## 4. Current Indications for Permanent Ostomies

As discussed earlier, several technological advances have significantly reduced the rate of APRs in rectal cancer patients, with the proportion of those undergoing APR decreasing from 31.8% in 1998 to 19.2% in 2011 [46]. However, APR remains the most appropriate option for patients with rectal cancer when an R0 resection with clear margins cannot be achieved without sphincter sacrifice, and where functional consequences of surgery are unavoidable. The general consensus is that patients with an invasive, cT2-4 rectal cancer who meet one of these criteria should be treated with an APR: a negative distal margin of 1 cm cannot be achieved with any of the sphincter-sparing procedures; locally advanced low-lying rectal cancer; locally recurrent low-lying rectal cancer, involvement of the external sphincter or invasion of the levator ani complex, and poor presurgical anorectal function [47,48,49]. The indications for permanent ostomy in rectal cancer are summarized in Table 1.

A thorough preoperative evaluation of baseline bowel function is critical to prevent subjecting patients to sphincter-preserving procedures that may result in suboptimal functional outcomes. Progressively more distal surgical anastomoses are associated with a significant decline in anorectal function, characterized by increased stool frequency or urgency, perianal irritation due to seepage, decreased stool and flatus discrimination, incomplete evacuation, and decreased rectal compliance [50]. While permanent abdominal stomas tend to alter routine physical and sexual activities and body image to varying degrees [51,52,53], patients who undergo APR avoid the common functional disorders associated with sphincter-preserving surgeries [51]. A few prospective studies demonstrated that low anastomoses with sphincter preservation were associated with significant fecal urgency and worse quality of life than patients with permanent ostomies [54,55], emphasizing the importance of preoperative counseling and surgical decision-making.

## 5. Current Indications for Temporary Diverting Ostomies

Diverting ostomies are created to divert the fecal stream and minimize the consequences of anastomotic leaks; both loop ileostomy and loop transverse colostomy are effective in serving this purpose [56,57,58]. Each type of ostomy has a distinct complication profile: loop colostomy is associated with higher rates of stoma prolapse, retraction, surgical site infection, and incisional hernia, while loop ileostomy is more commonly linked to dehydration and renal insufficiency [58]. Most surgeons prefer a loop ileostomy because of the ease of operative reversal. Loop colostomy is generally reserved for patients with pre-existing renal insufficiency or those for whom stoma reversal is unlikely [59].

The routine use of diverting ostomies after LAR is decreasing, and the approach is trending towards a selective one [11]. While patients without diverting ileostomies experience a higher incidence of anastomotic leaks compared to those with diversion (OR 0.292, 95% CI, 0.177–0.481), the rate of non-anastomotic complications is significantly greater in patients with diverting ileostomies than in those without (OR 3.377, 95% CI, 1.570–7.093) [60]. As it became evident that the benefits of routine diversion may not outweigh its short- and long-term morbidities [61,62,63,64], greater focus has been placed on identifying risk factors for anastomotic leaks for selective diversion.

Diversion is indicated for those with a high preoperative risk for anastomotic leak based on patient and technical factors. A systematic review of 23 studies examining the risk factors for anastomotic leak found that patients with low rectal anastomosis (OR-3.26, 95% CI, 2.31–462) male sex (OR = 1.48, 95% CI, 1.37–1.60, or preoperative radiotherapy (OR = 1.65, 95% CI, 1.06–2.56) may benefit most from fecal diversion [65]. The literature on the risk of anastomotic leak related to preoperative steroid use [66] is mixed. The most consistently identified technical risk factor for an anastomotic leak is the creation of low colorectal or coloanal anastomosis [65,67,68,69]. A low anastomosis at 5 cm from the anal verge had a five to six times increased odds of anastomotic leak after multivariate analysis [67,69].

A diverting ostomy can be considered in cases of obstructing distal tumors as a bridging operation, with the goal of eventually restoring bowel continuity. An ostomy can provide decompression and give the patient a chance to receive neoadjuvant therapy for an eventual elective resection [70,71]. A systematic review demonstrated that those who had diverting colostomy followed by resection had a higher chance of having continuity of bowel eventually restored than those who underwent primary resection with end colostomy in obstructing left-sided colon and rectal cancers (40% vs. 64%, *p* < 0.001) [70,71]. The indications for temporary diverting ostomy in rectal cancer are summarized in Table 1.

## 6. Complications and Morbidities of Ostomies

### 6.1. Non-Closure of Temporary Ostomies

Although diverting ostomies are intended to be temporary, around 20% either remain unclosed or are converted to permanent end colostomies [72,73]. This outcome is often attributed to complications such as anastomotic leaks or cancer recurrence [72,73,74]. Several preoperative risk factors associated with the likelihood of non-closure of temporary ostomies include advanced age, male sex, an ASA score ≥3, the presence of comorbidities, and distant metastases [73,75].

It is also important to note that permanent ostomies may serve as a last-resort option for managing major low anterior resection syndrome (LARS), a functional disorder with symptoms characterized by symptoms such as fecal incontinence, urgency, stool clustering, and stool fragmentation, often resulting in “toilet dependence” [76]. Common interventions for managing LARS include dietary modification, antidiarrheal medications, pelvic floor rehabilitation, transanal irrigation [77], and sacral neuromodulation [78]. However, for patients with severe, refractory symptoms, a permanent ostomy may be necessary [76]. It has been reported that around 6% may end up with a permanent ostomy due to unsatisfactory anorectal function from LARS [79,80].

### 6.2. Ostomy-Related Complications

Stomal complications are diverse and can significantly impact patient outcomes and quality of life. An ileostomy is associated with physiological changes; the all-cause 30-day readmission rate following an ileostomy is approximately 30%, with dehydration from high-output ileostomy accounting for 40% of these cases [81,82]. Parastomal hernias, which occur in up to 40% of all end colostomy patients, may necessitate reoperation in cases of strangulation and bowel ischemia [83] or result in chronic symptoms that severely impair quality of life [84]. Stomal prolapse, occurring in 7 to 26% of ostomies, is more common in loop transverse colostomies [85]. Stomal prolapse and stomal stenosis may lead to complications requiring surgical interventions, such as bowel resection and stoma relocation [86]. Parastomal skin complications are also prevalent, occurring in up to 43% of cases, particularly with ileostomies [87]. These complications often result from chemical injuries from effluent leakage, mucocutaneous separation, mechanical trauma from appliance use, contact dermatitis, or pyoderma gangrenosum [85,87].

### 6.3. Complications Associated with Closure of Ostomies

The closure of diverting ostomies carries a complication rate of approximately 11–20%, with common issues including small bowel obstruction, wound infection, anastomotic leak, and enterocutaneous fistula [88,89,90]. A complication gaining increased attention is the development of incisional hernias at the reversal site, which occur in roughly 20% of cases following loop ileostomy reversal and within 2 years [91,92,93]. This growing awareness underscores the need for careful surgical planning and counseling regarding potential risks associated with ostomy closure.

## 7. Strategies to Mitigate Ostomy-Related Complications

### 7.1. Technical Considerations at the Time of the Operation

Several important considerations must be addressed preoperatively, with one of the most critical being the proper marking of a stoma site or multiple sites if the operative plan remains uncertain. The patient should be evaluated for marking in various positions, including supine, sitting up, and standing [85]. The chosen stoma site should be positioned away from bony prominences, planned or previous incisions, and natural skin creases [85]. Ideally, it should be surrounded by at least a 2-inch area of flat, healthy skin to ensure a reliable seal for the appliance [85,94]. Proper stoma site selection is essential for effective pouching that minimizes the risk of peristomal skin complications.

Vascular compromise of the stoma can lead to complications such as ischemia, necrosis, and stenosis. To minimize the risk, excessive trimming of the epiploic fat and the mesentery and excess tension should be avoided. For an ileostomy, maintaining adequate blood supply typically requires preserving the mesentery within 5 cm of the distal end [95]. In the case of a colostomy, at least 1 cm of the colonic mesentery adjacent to the bowel wall should be preserved to ensure the patency of the marginal artery [95].

### 7.2. Techniques to Mitigate Parastomal Hernias

Several techniques have been proposed to reduce the risk of future parastomal hernias during the surgery. One such approach is the use of an extraperitoneal route for end colostomy, where the colon is tunneled between the peritoneum and the abdominal wall muscles. A meta-analysis of two randomized controlled trials (RCTs) and eight retrospective studies demonstrated that extraperitoneal colostomy significantly reduced parastomal hernia rates compared to transperitoneal colostomy (6.3% vs. 17.8%, *p* < 0.001) (risk ratio −0.36, 95% CI, 0.21–0.62, I2 = 26%) [96]. However, the extraperitoneal approach may be less practical during laparoscopic surgery, necessitating the development of new laparoscopic techniques [97,98].

Since the 2000s, increasing research has focused on the use of prophylactic, either nonabsorbable keyhole synthetic [99,100,101], biosynthetic [102], or biologic [103] meshes to prevent parastomal hernias. Several meta-analyses have compared the outcomes using prophylactic mesh (both biologic and synthetic) versus no mesh at the time of colostomy creation [104,105,106,107]. Although most meta-analyses showed that prophylactic mesh reduces the incidence of parastomal hernias without increasing colostomy-specific complications or morbidity [104,105,106], one recent meta-analysis found no clear benefit when only studies with follow-ups exceeding two years were analyzed [107]. The Chimney Trial in 2024 compared parastomal hernia rates between a no-mesh group and a group that received prophylactic funnel-shaped synthetic mesh specifically designed for parastomal use [108]. After 12 months, CT-confirmed parastomal hernia occurred in 10% of the intervention group vs. 37% in the control group, representing a 27% absolute risk reduction (95% CI, 12–41; *p* < 0.001) [108].

As an end colostomy is created for permanence, challenges of balancing reinforcement with maintaining stoma function and preventing stoma-related complications continue to be the focus of research in this area.

### 7.3. Techniques to Mitigate Incisional Hernias After Ostomy Reversal

Unlike parastomal hernias, which primarily occur with end colostomies, strategies to reduce incisional hernias using prophylactic mesh have been applied to both ileostomy and colostomy reversals. Numerous studies have explored various mesh types, such as non-absorbable [109,110], absorbable [111], or biological [112,113] materials, as well as different placement techniques, including onlay [110], retrorectus [114], and underlay [109] repairs. Several meta-analyses have demonstrated that prophylactic mesh use, regardless of the type or location, is associated with a reduced incidence of stoma site incisional hernias without increasing the risk of surgical site infection or length of stay [105,115,116]. Notably, one meta-analysis found no significant superiority of any specific mesh type [115].

**Figure 1:**
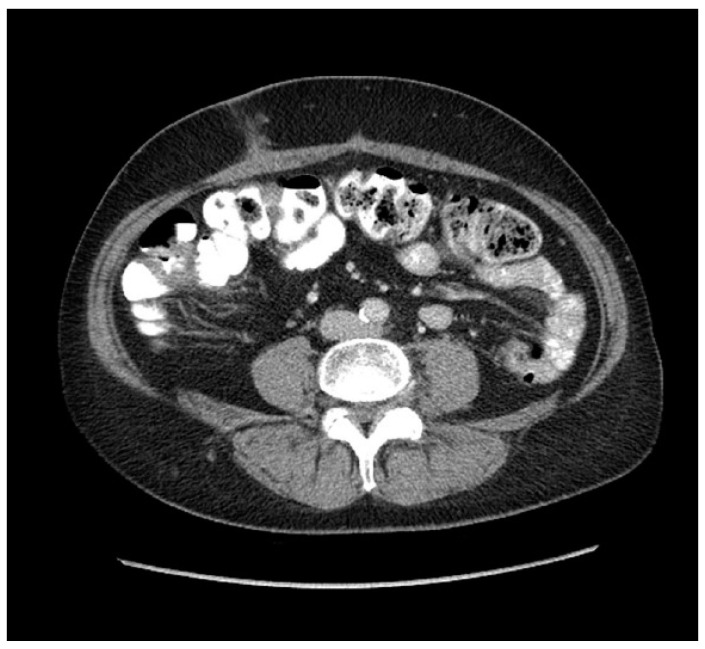
Surveillance computed tomography image after a prophylactic retrorectus synthetic mesh repair at the time of diverting ileostomy reversal.

One of the most notable large RCTs in recent years is the multicenter Reinforcement of Closure of Stoma Site (ROCSS) RCT, which included 790 patients to compare the outcomes of biological mesh reinforcement with suture-only repair [117]. At a 2-year follow-up, the incidence of incisional hernias was significantly lower in the mesh group compared to the control group (12% vs. 20%), with an adjusted relative risk of 0.62 (95% CI, 0.43–0.90, *p* = 0.012) [117]. However, this approach has not gained widespread popularity due to the high cost of biologic mesh and the challenges associated with intra-abdominal underlay placement. Retrorectus repair using synthetic mesh has shown promising results in individual studies. A prospective Stoma Closure And Reinforcement (SCAR) phase I/II trial, which enrolled 20 patients undergoing ileostomy reversal with retrorectus polypropylene mesh reinforcement, reported no surgical site infection or hernias after a 20-month follow-up period [114]. Figure 1 shows an interval surveillance computed tomography image of a prophylactic retrorectus synthetic mesh stoma site hernia repair at the time of diverting loop ileostomy reversal, demonstrating a subcutaneous scar without evidence of fascial disruption from the SCAR trial [114].

## 8. Conclusions

In summary, the evolution of rectal cancer surgery has been marked by significant advancements aimed at improving oncological outcomes and preserving patient quality of life. Innovations, such as accurate and reliable imaging, neoadjuvant therapy, and minimally invasive techniques have improved survival rates and minimized the need for both permanent and temporary ostomies. Permanent ostomies remain necessary in specific cases, such as advanced distal rectal cancer or impaired sphincter function. Temporary diverting ostomies are critical in high-risk patients or for protecting low anastomosis. Despite their benefits, ostomies are associated with a range of complications, including hernias, prolapse, skin complications, incisional hernia, and the psychosocial burden they impose on patients. Understanding the indications and associated risks of ostomies is essential for personalized surgical planning and optimizing outcomes. Continued innovation in surgical techniques and adjuvant therapies will be pivotal in optimizing the appropriate use of ostomies and reducing their associated complications.

## Figures and Tables

**Table 1:** Current indications for ostomies in rectal cancer surgery.

Permanent Ostomies	Temporary Diverting Ostomies
Inability to achieve a negative distal margin of 1 cm with sphincter-sparing procedures Locally advanced or recurrent low-lying rectal cancer Involvement of the external sphincter or invasion of the levator ani complex Poor presurgical anorectal function	Patients at high risk for anastomotic leaks, including co-morbidities, preoperative radiation, and immunosuppression Low anastomosis at 5 cm from the anal verge Obstructing distal tumors: for symptomatic relief or as a bridging operation for a definitive surgery
